# Severe Acute Respiratory Syndrome Coronavirus 2 in Farmed Mink (*Neovison vison*), Poland

**DOI:** 10.3201/eid2709.210286

**Published:** 2021-09

**Authors:** Lukasz Rabalski, Maciej Kosinski, Teemu Smura, Kirsi Aaltonen, Ravi Kant, Tarja Sironen, Bogusław Szewczyk, Maciej Grzybek

**Affiliations:** University of Gdansk, Gdansk, Poland (L. Rabalski, M. Kosinski, B. Szewczyk);; University of Helsinki, Helsinki, Finland (T. Smura, K. Aaltonen, R. Kant, T. Sironen);; Medical University of Gdansk, Gdansk, Poland (M. Grzybek)

**Keywords:** severe acute respiratory syndrome coronavirus 2, SARS-CoV-2, coronaviruses, viruses, coronavirus disease, COVID-19, respiratory infections, mink, Neovison vison, transmission, spillover, detection, molecular characterization, zoonoses, Poland

## Abstract

Severe acute respiratory syndrome coronavirus 2 (SARS-CoV-2) is the etiologic agent of coronavirus disease and has been spreading worldwide since December 2019. The virus can infect different animal species under experimental conditions, and mink on fur farms in Europe and other areas are susceptible to SARS-CoV-2 infection. We investigated SARS-CoV-2 infection in 91 mink from a farm in northern Poland. Using reverse transcription PCR, antigen detection, and next-generation sequencing, we confirmed that 15 animals were positive for SARS-CoV-2. We verified this finding by sequencing full viral genomes and confirmed a virus variant that has sporadic mutations through the full genome sequence in the spike protein (G75V and C1247F). We were unable to find other SARS-CoV-2 sequences simultaneously containing these 2 mutations. Country-scale monitoring by veterinary inspection should be implemented to detect SARS-CoV-2 in other mink farms.

Identifying possible pathogen hosts and studying transmission dynamics of hosts in their populations are crucial steps in controlling zoonotic diseases. The origin of severe acute respiratory syndrome coronavirus 2 (SARS-CoV-2) is probably bats (*1*), but the potential intermediate host has not yet been confirmed. SARS-CoV-2 seems to readily jump from humans to other animal species, particularly carnivores (i.e., dogs, cats, ferrets, lions, pumas) (*2*,*3*), raising concerns about new animal sources of coronavirus disease (COVID-19) (*4*,*5*).

SARS-CoV-2 infections in mink were reported from farms in Denmark and the Netherlands and later in other regions (*6*–*9*) (Figure 1). Because of SARS-CoV-2 outbreaks in mink farms and their appearance in the surrounding communities, the European Centre for Disease Prevention and Control and the World Health Organization have emphasized the need for surveying the host‒animal interface by collaboration among virologists and epidemiologists to track and characterize viral mutations (*11*). After SARS-CoV-2 infections in mink in the Netherlands, the Dutch Ministry of Agriculture decided to cull the mink from all farms. In Denmark, the Danish National Institute of Public Health announced the culling of all 17 million mink in the country after the virus had passed back from the mink farms into the human community. The data available for Denmark on these mink-associated SARS-CoV-2 variants suggest that these variants can spread rapidly on mink farms and in nearby human communities (*12*). However, humans infected with the mink-related variants do not appear to have more severe clinical symptoms than those infected with non–mink-related variants (*9*).

**Figure 1:**
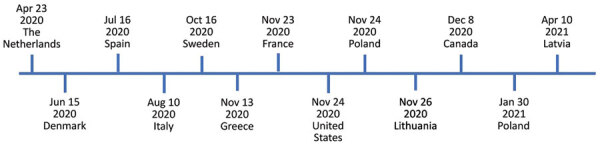
Timeline of severe acute respiratory syndrome coronavirus 2 (SARS-CoV-2) infections in mink farms, Europe, according to the World Organisation for Animal Health (*10*). We investigated SARS-CoV-2 in mink sampled on November 24, 2020, in Pomorskie Voivodeship, northern Poland. The Polish National Veterinary Research Institute, as a national unit responsible for reporting to the World Organisation for Animal Health, detected SARS-CoV-2 infection in the same mink farm on January 30, 2021.

Poland is the second-largest producer of mink pelts in Europe. Poland has 810 fur animal farms, including those for foxes, mink, raccoons, dogs, and chinchillas. The 354 active mink farms in Poland contain ≈6.3 million mink. During 2019, mink farmers in Poland sold 8.5 million mink pelts (*13*,*14*).

As of May 5, 2021, Poland had recorded 2,838,180 COVID-19 cases and 70,336 total related deaths (*15*). Considering the recent reports of SARS-CoV-2 infections in mink in other countries in Europe and the high incidence of human SARS-CoV-2 infections in Poland, we monitored SARS-CoV-2 in mink on 1 farm in Pomorskie Voivodeship in northern Poland.

## Materials and Methods

### Material Collection

We collected throat swab (BIOCOMA, http://www.biocomma.com) specimens from 91 mink culled for pelting at a mink farm in Pomorskie Voivodeship in northern Poland on November 17, 2020. The farm owner reported no respiratory symptoms in the animals. We collected blood samples directly from the heart by using cardiac puncture and a sterile 5-mL syringe immediately after death of the mink. After separation of the blood clot, we centrifuged samples at 5,000 rpm for 10 min by using an MPW High-Speed Brushless Centrifuge (MPW Med Instruments, https://mpw.pl). We collected serum and stored it at −80°C until the samples could be analyzed.

### RNA Isolation

We added 150 μL of each swab specimen sample in inactivation buffer to 300 μL of RLT lysis buffer (RNeasy Mini Kit; QIAGEN, https://www.qiagen.com). We then mixed samples vortexing and incubated them for 10 min at room temperature. After incubation, we added 400 μL of 70% ethanol to each sample and mixed them by pipetting. We transferred lysates to RNeasy Mini spin columns with collection tubes (QIAGEN) and centrifuged them at 13,000 rpm for 1 min. We then washed the columns once with 700 μL of RW1 buffer and twice with 500 μL of RPE buffer. Between every wash, we centrifuged the columns and discarded the flow-through. We performed elution by adding 50 μL of PCR-grade water to the columns and incubating them for 2 min. We placed the columns into new tubes and centrifuged them at 13,000 rpm for 1 min. After isolation, we stored the samples for <2 hours at 4°C. No human-origin samples were processed at the same time.

### SARS-CoV-2 Case Definition

We defined SARS-CoV-2‒positive animals as suggested by the World Organisation for Animal Health (*10*). We considered mink to be SARS-CoV-2 positive if SARS-CoV-2 was isolated from a sample taken directly from an animal (nasal or oropharyngeal swab sample) or if viral nucleic acid was identified in a sample taken directly from an animal, giving cause for suspicion of previous association or contact with SARS-CoV-2, by targeting >2 specific genomic regions at a level indicating presence of infectious virus or by targeting 1 genomic region, followed by sequencing of a secondary target.

### Real-Time Reverse Transcription PCR

For each sample, we prepared the reaction mixture by using a TaqPath 1-Step quantitative RT-PCR (reverse transcription PCR) Master Mix (ThermoFisher Scientific, https://www.thermofisher.com), polymerase, diethyl pyrocarbonate‒treated water (EURx, https://eurx.com.pl) and primers and probes for the RNA-dependent RNA polymerase (RdRp) and envelope (E) genes (*16*) in white, 8-well, quantitative PCR strips with optical clear caps (Applied Biosystems, https://www.thermofisher.com). We also prepared positive control plasmids made in-house containing the RdRp and E genes and a no-template control containing diethyl pyrocarbonate‒treated water instead of template reactions. We mixed reactions and loaded them into a Light Cycler 480 (Applied Biosystems, https://www.thermofisher.com). Cycling conditions were incubation with uracil N-glycosylase for 2 min at 25°C, reverse transcription for 15 min at 50°C, and enzyme activation for 2 min at 95°C, followed by 40 amplification cycles consisting of 3 s at 95°C and 30 s at 60°C. After each amplification cycle, we measured the signal from each sample in both the FAM (RdRp gene) and HEX (E gene) channels. Samples with a crossing point (Cp) <35 for any gene were considered positive for SARS-CoV-2.

### SARS-CoV-2 Antigen Detection in Mink

We performed 2 different antigen tests to confirm the presence of viral antigen in either the swab or serum samples. Antigen tests were conducted on samples positive by quantitative PCR. We used 3 negative samples from the same batch as the positive samples as controls. A total of 150 μL of transport medium from each swab specimen was transferred to tubes from a COVID-19 Antigen Detection Kit (Zhuhai Lituo Biotechnology Co., Ltd., https://www.lituo.com.cn) containing extraction buffer. This antigen test was in the form of a cassette with a lateral flow assay that targets the nucleocapsid protein of SARS-CoV-2. Samples were mixed and incubated for 1 min at room temperature. We added 2 drops of each sample to the sample window on the test cassettes. Results consisted of 2 bands: 1 for the control and 1 for the target. If both bands showed a burgundy line, the test result was considered positive. We read results after 12 min.

We tested 91 mink serum samples by using a SARS-CoV-2 antigen ELISA (COV-04-S; Salofa Oy, https://www.salofa.com) according to the kit instructions. This test is a double-antibody sandwich ELISA. The results were obtained according to the formula based on the concentration standards provided in the kit. The cutoff value for this test was 2.97 pg/mL. The tests were repeated twice, and additional dilutions were performed to determine the final concentration as suggested in the kit instructions.

### Full SARS-CoV-2 Genome Sequencing and Classification

We performed SARS-CoV-2 genome sequencing at University of Gdansk in Poland and the University of Helsinki in Finland by using only samples containing RNA isolated from virus-positive swab specimens (amplification of target gene in an RT-PCR; this gene has a Cp <35) or that were inconclusive (only 1 target gene amplification with Cp <35). Samples with positive results in the SARS-CoV-2 antigen detection assays were also sequenced.

At Gdansk, 2 independent protocols were used for SARS-CoV-2 genome sequencing. The first protocol was an Illumina (https://www.illumina.com) RNA preparation with enrichment for respiratory virus oligos panel V2, followed by an Illumina MiniSeq medium output run that produced 150-nt paired-end reads. The second protocol was an ARTIC version 3 amplicon generation (https://bmcgenomics.biomedcentral.com), followed by an Oxford Nanopore Technology MinIONB run (https://nanoporetech.com). No human origin samples were processed at the same time. No DNA/rRNA depletion methods were used. Reads were base called, de-barcoded, and trimmed to remove adaptor, barcode, and PCR primer sequences. Oxford Nanopore Technology reads were used for SARS-CoV-2 genome assembly in ARTIC-nCoV-bioinformaticsSOP-v1.1.0 (https://artic.network/ncov-2019). 

Illumina paired reads were used to prepare contigs by de novo assembly by using the Geneious Prime 2020.2 (https://www.geneious.com) software suite with integrated tools for variant calling (minimum coverage = 100, minimum variant frequency = 0.25) and consensus sequence generation. The fasta files generated by the Illumina procedure were further analyzed in Kraken2 2.1.1 software (https://www.ccb.jhu.edu/software/kraken2) to classify every read to the reference database containing viral and American mink genomes (*17*).

In Helsinki, the sequencing libraries were prepared by using the Illumina DNA Prep Kit (New England BioLabs, https://www.neb.com). We measured library fragment sizes by using agarose gel electrophoresis and concentrations by using the Qubit dsDNA HS Assay Kit (Life Technologies, https://www.thermofisher.com) and the NEBNext Library Quant Kit for Illumina (New England BioLabs). Sequencing was conducted by using the MiSeq V3 Reagent Kit (Illumina) with 250-bp reads. We trimmed raw sequence reads and removed low-quality (quality score <30) and short (<50 nt) sequences by using Trimmomatic (*18*). Trimmed sequence reads were mapped against the SARS-CoV-2 reference sequence (GenBank accession no. NC_045512.2) by using BWA-MEM version 0.7.17 (*19*), followed by sorting and removal of duplicate reads by using SAMTools version 1.10 (*20*).

### Phylogenetic Analysis of SARS-CoV-2 Isolates

The dataset consisted of all genetic sequences of SARS-CoV-2 from this study (Poland, Germany, Lithuania, Latvia, Estonia, Russia, and Ukraine), which was completed as the representative pool Europe by Nexstrain (https://nextstrain.org/ncov/europe) and resulted in 5,778 entries. We performed phylogenetic analysis by using the procedure recommended by Nextstrain with modifications in the subsampling region filtering procedure, in which the number of sequences per country was 40 (*21*). We used Augur toolkit version 10.1.1 (Nextstrain) for phylogenetic analysis and Auspice version 2.10.1 (Nextstrain) for visualization. Possible time of divergence for samples was inferred by using the TreeTime pipeline (https://www.treetime.com) implemented in the Nextstrain analysis and presented in the phylogenetic tree (*22*).

### Statistical Analysis and Ethics

We calculated 95% CIs by using published procedures (*23**,**24*). This study was conducted with due regard for European Union Principles and the Polish Law on Animal Protection. No permit from the Local Bioethical Committee for Animal Experimentation was obtained because animals were culled by the owner for production of pelts. Samples were collected postmortem.

## Results

### Prevalence of SARS-CoV-2

We confirmed that 15 mink (16.5%, 95% CI 8.4%–28.6%) were positive for SARS-CoV-2. We summarized and provide the diagnostic results (Table 1).

**Table 1:** Detection of SARS-CoV-2 by different techniques in samples collected from 19 farmed mink, Poland*

Sample name	Cp for RdRP gene†	Cp for E gene†	Antigen in swab specimen	Antigen in serum, concentration of N protein, pg/mL	Sequence obtained
**mink_­4**	**ND**	**24.90**	**Strong positive**	**46.86**	**Full**
**mink_­5**	**24.36**	**16.88**	**Strong positive**	**3.30**	**Full**
**mink_­6**	**ND**	**34.88**	**Negative**	**ND**	**Partial**
mink_­20	ND	ND	Positive	132.24	Partial
**mink_27**	**ND**	**35.06**	**Negative**	**ND**	**Partial**
mink_­36	ND	ND	Positive	271.30	Partial
mink_39	ND	ND	Negative	35.59	Partial
**mink_­42**	**ND**	**25.62**	**Positive**	**ND**	**Full**
**mink_­46**	**34.93**	**22.75**	**Positive**	**ND**	**Full**
**mink_­48**	**29.89**	**22.52**	**Strong positive**	**ND**	**Full**
**mink_­49**	**ND**	**27.79**	**Positive**	**ND**	**Full**
**mink_­50**	**32.39**	**21.61**	**Strong positive**	**ND**	**Full**
mink_­63	‒	‒	Negative	19.30	Partial
**mink_­67**	**36.88**	**23.34**	**Positive**	**ND**	**Full**
**mink_­70**	**ND**	**24.40**	**Positive**	**ND**	**Partial**
**mink_­76**	**32.53**	**22.73**	**Positive**	**ND**	**Full**
**mink_­77**	**32.87**	**20.30**	**Strong positive**	**ND**	**Full**
**mink_­83**	**15.39**	**21.77**	**Strong positive**	**91.57**	**Full**
**mink_­88**	**ND**	**33.99**	**Positive**	**ND**	**Full**
NTC	ND	ND	ND	ND	ND
Positive control	16.91	23.31	ND	ND	ND

*Samples in bold are considered positive for SARS-CoV-2 by nucleic acid amplification, serologic analysis, or next-generation sequencing. Cp, crossing point; E, envelope; N, nucleocapsid; ND, not detected; NTC, no template control; RdRp, RNA-dependent RNA polymerase; SARS-CoV-2, severe acute respiratory coronavirus 2. †By reverse transcription quantitative PCR.

### SARS-CoV-2 Antigen Detection in Mink

Samples mink_4, mink_5, mink_48, mink_50, mink_77, and mink_83 had highly visible signals in both the control and test lines. Samples mink_20, mink_36, mink_42, mink_46, mink_49, mink_67, mink_76, and mink_88 had a highly visible control line and a much less pronounced test line. In all other samples, only the test line was visible. All 8 real-time RT-PCR‒positive samples were also positive by the antigen test. In addition, 5 E gene‒positive samples were also positive in the antigen test, but 4 were negative. The sample from mink_20 was positive in the antigen test, but SARS-CoV-2 RNA was not detected by RT-PCR.

### Read Classification and SARS-CoV-2 Genome Sequences

The final validation of detection of SARS-CoV-2 in the mink was classification of the next-generation sequencing reads on the basis of the database containing reference viral, human, and American mink genomes. We used 3 independent approaches to obtain full viral genomes (Table 2). Only samples that had a complete SARS-CoV-2 genome sequence are shown. The number of Illumina reads generated for samples mink_4, mink_42, mink_49, mink_76, and mink_88 was not enough to produce full SARS-CoV-2 genomes. For these samples, the genomes were obtained by using the ARTIC procedure.

**Table 2:** Total mean coverage for each sample from 12 mink with a full SARS-CoV-2 genome, Poland*

Sample name	Mean coverage of SARS-CoV-2 genome (ONT plus Illumina)	Total Illumina reads†	Total Illumina SARS-CoV-2 reads†	Total Illumina mink reads†
mink_4	949.6	152,609	37	131,204
mink_5	2,776	396,259	159,484	184,135
mink_42	847.8	66,515	868	34,968
mink_46	1,039.5	387,682	25,975	241,788
mink_48	1,151	125,328	9,526	64,363
mink_49	394.6	487,771	1,711	348,017
mink_50	1,839.6	425,333	79,589	246,546
mink_67	882.1	167,963	10,834	114,163
mink_76	723.0	99,660	2,105	77,945
mink_77	16,078.5	2,424,311	1,609,172	662,381
mink_83	111,978.9	13,495,934	12,730,431	611,926
mink_88	1,205.2	1,071,003	2,822	611,787

*ONT, Oxford Nanopore Technology (https://nanoporetech.com); SARS-CoV-2, severe acute respiratory syndrome coronavirus 2. †These columns indicate how many Illumina (https://www.illumina.com) reads were generated for each sample and how many of them were classified as SARS-CoV-2 or American mink genomes.

### Phylogenetic Analysis of SARS-CoV-2 in Farmed Mink

**Figure 2:**
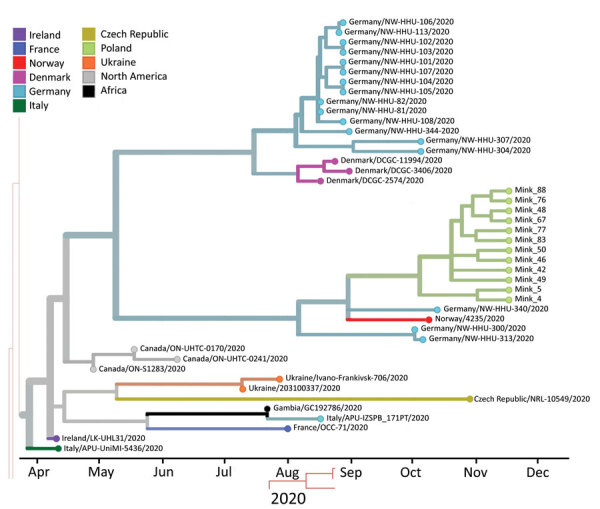
Phylogenetic tree estimating the divergence time for severe acute respiratory syndrome coronavirus 2, Europe. Sequences from mink sampled on November 24, 2020, in Pomorskie Voivodeship, northern Poland, are shown on green branches. Only closely related isolates that were included in the dataset are presented. Visualization was achieved by using Nextstrain (https://nextstrain.org/ncov/europe).

We checked the 12 mink-originated SARS-CoV-2 sequences for mink-specific mutations detected earlier in mink from the Netherlands and Denmark, but found none. This finding suggested recent and separate introduction of SARS-CoV2 into mink from Poland (Figure 2). Alignment of full-genome sequences from 12 samples showed multiple polymorphisms at different nucleotide sites. Many of these polymorphisms gave rise to changes in amino acids when compared with the reference Wuhan-Hu-1 sequence (GenBank accession no. MN908947). Two specific mutations present in all samples were found in the spike protein: G75V and C1247F. The G75V mutation is present in 199 isolates published in GISAID (https://www.gisaid.org), and the C1257F mutation in 83 isolates. Other rare amino acid variants present in every SARS-CoV-2 isolate from mink in Poland were found in 5 additional proteins: nonstructural protein (nsp) 2, nsp3, nsp14, nsp15, and nucleocapsid protein.

On the basis of the dataset, we inferred a phylogenetic relationship by estimating the divergence times between each isolate (Figure 2). The analysis estimated that the most recent common ancestor for SARS-CoV-2 from mink in Poland and the 2 most similar sequences (German/NW-HHU-340/2020 and Norway/4235/2020) diverged on approximately September 31, 2020. We recognized mutations in amino acid sequences. If the molecular evolution started after virus introduction into the farm, this incident is estimated to have occurred on approximately October 4, 2020. Complete genome sequences of SARS-CoV-2 isolated from farmed mink in Poland have been deposited in GISAID (accession nos. EPI_ISL_732948‒59).

## Discussion

Identifying new species that can serve as animal sources of SARS-CoV-2 and predicting where novel outbreaks are most likely to occur are crucial steps for preventing and minimizing the extent of SARS-CoV-2 infections among humans (*25*). Recent reports confirmed the presence of SARS-CoV-2 in different animal species, including fur animals (i.e., mink and racoon dogs) (*26*,*27*). We report a 16.5% prevalence of SARS-CoV-2 in mink tested at a fur farm in northern Poland, confirming the presence of SARS-CoV-2 in farmed mink in Poland.

During our study, we used 2 different sequencing technologies to sequence the SARS-CoV-2 genomes. We found that amplicon-based nanopore sequencing gave better results than the bed-based enrichment Illumina approach. Conversely, Illumina reads showed a broader context because we were able to classify background reads that do not map to the SARS-CoV-2 genome as being of host origin. Therefore, these reads can be used as proof of sample origin. We also showed that the RdRp target for the quantitative PCR is less effective than the E gene in our experiment settings. The full genome of SARS-CoV-2 was assembled when both target genes were detected or the E gene was detected by a pair of positive signals in the antigenic assay.

Poland is one of the largest fur producers in Europe. Considering the number of farmed mink in the country and the large number of persons employed in this sector, we seek to increase awareness in the scientific community and mink industry that mink are susceptible to SARS-CoV-2 infection. Previous studies reported viral RNA detection in airborne inhalable dust in mink farms (*8*). Moreover, close contact of farmworkers with animals during feeding, culling, and dehiding increases their risk for exposure. We believe that a country-scale biomonitoring program should be activated as soon as possible to prevent the fur production sector from being a reservoir for future spillover of SARS-CoV-2 to humans. Samples for molecular diagnostics should be obtained from all farms in Poland following the highest standards for material collection, sample handling, and molecular detection of SARS-CoV-2.

We report a possible new genotype of SARS-CoV-2 that has sporadic mutations throughout the full genome sequence. Two mutations located in the spike protein (G75V and C1247F) were present in all isolates reported in this study. The G75V mutation is localized in the N terminal domain and could be responsible for interactions with host receptors or stabilizing the spike protein in a constrained prefusion state (*28*). To date, no other SARS-CoV-2 sequences deposited in GISAID have these 2 mutations simultaneously (*29*). We have recently detected possible zoonotic spillover of SARS-CoV-2 in worker employed at the farm described in this study (L. Rabalski et al., unpub. data). Preliminary genome analysis showed that the newly described isolates carry the combination of mutations typical of viruses isolated in November 2020, but additional new changes have accumulated since that time. We believe that wide monitoring of humans living near the mink farm should be performed to search for possible spillover and presence of new virus variants. Constant epizootiology monitoring is a crucial step in preventing new outbreaks of zoonotic diseases.
