# Correction: Vol. 27, No. 9

**DOI:** 10.3201/eid2801.C12801

**Published:** 2022-01

**Keywords:** errata, erratum, correction

The acknowledgments and funding information were inaccurate in Severe Acute Respiratory Syndrome Coronavirus 2 in Farmed Mink (*Neovison vison*), Poland (L. Rabalski et al.). The article has been corrected online (https://wwwnc.cdc.gov/eid/article/27/9/21-0286_article).

